# Comparison of commercially available three‐dimensional treatment planning algorithms for monitor unit calculations in the presence of heterogeneities

**DOI:** 10.1120/jacmp.v2i1.2625

**Published:** 2001-01-01

**Authors:** Joseph R. Butts, Alvis E. Foster

**Affiliations:** ^1^ Ball Memorial Hospital, Radiation Oncology Department 2401 W. University Avenue Muncie Indiana 47303

**Keywords:** radiation therapy, monitor units, treatment planning algorithm, heterogeneity corrections

## Abstract

This study uses an anthropomorphic phantom and its computed tomography (CT) data set to evaluate monitor unit (MU) calculations using the CMS Focus Clarkson, the CMS Focus Multigrid Superposition Model, the CMS Focus FFT Convolution Model, and the ADAC Pinnacle^3^ Collapsed Cone Convolution Superposition Algorithms. Using heterogeneity corrections, a treatment plan and corresponding MU calculations were generated for several typical clinical situations. A diode detector, placed in an anthropomorphic phantom, was used to compare the treatment planning algorithms' predicted doses with measured data. Differences between diode measurements and the algorithms' calculations were within reasonable levels of acceptability as recommended by Van Dyk *et al*. [Int. J. Rad. Onc. Biol. Phys. **26**, 261–273 (1993)], except for the CMS Clarkson algorithm, which predicted too few MU for delivery of the intended dose to chest wall fields.

PACS number(s): 87.53.Bn, 87.53.Dq

## I. Introduction

Several treatment planning algorithms are available commercially for calculating dose distributions from external photon beams. The accuracy with which these algorithms are able to predict dose is dependent upon the assumptions and approximations that the algorithm makes. Early methods^1^ ^–^ ^5^ corrected dose measurements obtained in a water phantom. More modern advances in computing power allowed the development of model‐based algorithms, which attempt to model from first principles the photon beam and its interaction with the patient.^6^ ^–^ ^18^ The commercially available algorithms are not distinctly categorized as correction‐based or model‐based algorithms, but rather fall somewhere along a spectrum. The objective of this work is to compare the implementations of several commercially available algorithms (Focus, Computerized Medical Systems, St. Louis MO, and Pinnacle^3^, ADAC Laboratories, Milpitas, CA) in their ability to predict monitor units under a variety of clinical situations, using heterogeneity corrections.

## II. Methods and Materials

### A. Diode detector

A cubic (30 cm×30 cm×30 cm) water phantom was set up to evaluate the characteristics of a 7.1‐mm diameter diode detector (ISORAD™, Sun Nuclear Corp., Melbourne, FL). By comparing percent depth dose measurements made with the diode detector with calculated percent depth doses, the diode's depth dependence was calibrated. Coupled with an electrometer (CNMC‐Model 206, CNMC Company, Inc., Nashville, TN), measurements were made in the photovoltaic mode along the central axis of 4 and 10 MV photon beams from one linear accelerator (Clinac 2100C, Varian Medical Systems, Palo Alto, CA) and along the central axis of 6 and 18 MV photon beams from another accelerator (Clinac 1800, Varian Medical Systems, Palo Alto, CA)

### B. Clinical geometry

A treatment planning CT was acquired for an anthropomorphic phantom (RANDO, Alderson Research Laboratories, Stamford, CT) in a supine position. Axial slices of 5 mm thickness were acquired from the top of the head to the middle of the femur. The images were transferred to the treatment planning systems and grouped according to four clinical sites: pelvis, head and neck, lung, and chest wall.

Beams were placed onto each site for the generation of monitor units. Each of the fields listed in Table I was designed to deliver 100 cGy to its isocenter. Placement of each isocenter is illustrated in Figs. 2–6. Calculations were performed using the CMS Clarkson, the CMS Fast‐Fourier Transform (FFT) Convolution, the CMS Multigrid Superposition (MGS), the CMS Fast MultiGrid Superposition, and the ADAC Collapsed Cone Convolution and Adaptive Convolution Algorithms, with a grid resolution of 0.4 cm.

**Figure 1:**
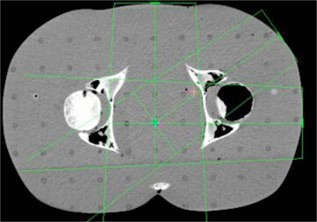
(Color) Pelvis setup.

**Figure 2:**
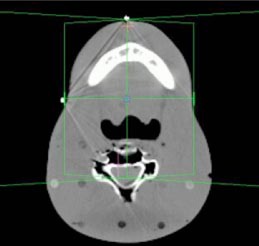
(Color) Head and neck setup. The isocenter is anterior to the air heterogeneity.

**Figure 3:**
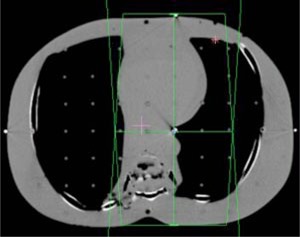
(Color) AP/PA lung setup. The isocenter is located at the lung/tissue interface.

**Figure 4:**
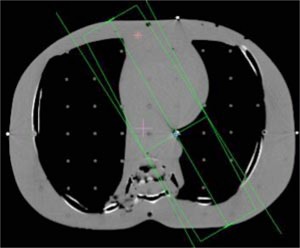
(Color) Oblique lung setup. The isocenter is located at the lung/tissue interface, with almost no buildup on the posterior beam.

**Figure 5:**
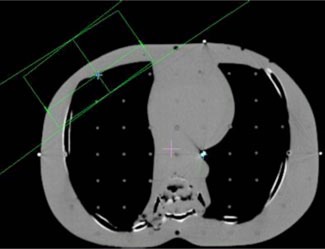
(Color) Chestwall setup. The isocenter is at the lung/tissue interface, with some flash over the phantom.

**Figure 6:**
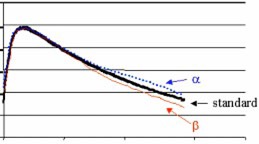
(Color) Potential modeling differences when commissioning treatment planning algorithms.

**Table I:** Clinical beams to be used.

Site	Beam direction	Field size	Energies
Pelvis	AP	10×8	10 MV, 18 MV
Pelvis	LT LAT	9×8	10 MV, 18 MV
Pelvis	LAO	8×8	10 MV, 18 MV
Head and Neck	RT LAT	12×9	4 MV, 6 MV
Lung	AP	13×15	6 MV, 10 MV
Lung	PA	13×15	6 MV, 10 MV
Lung	RAO	8×15	6 MV, 10 MV
Lung	LPO	8×15	6 MV, 10 MV
Chest Wall	Medial	7.5×16	4 MV, 6 MV

### C. Dose calculation algorithms

The CMS Clarkson algorithm is a measurement‐based method which uses a modified sector integration method based on tissue‐phantom (TPR) data generated from measured percent depth doses.^24^ Missing tissue outside the patient is unaccounted for. In addition, the algorithm assumes a homogeneous patient for scatter calculations.

The Focus Fast‐Fourier Transform (FFT) Convolution algorithm and the MultiGrid Superposition (MGS) algorithms use convolution/superposition methods^8^ ^–^ ^11^ to account for the transport of primary and secondary radiation inside the patient and to account for the effects of tissue heterogeneities. Both compute dose by convolving the total energy released per unit mass (TERMA) with Monte Carlo generated energy deposition kernels.^21^ The FFT algorithm speeds up its calculation by calculating dose in the frequency domain,^22^ while assuming kernels to be invariant with position. This technique ignores effects of heterogeneities on laterally scattered radiation. Conversely, kernels used for the MGS algorithm vary with position. To model heterogeneities, these kernels also vary with electron density, based on the electron density scaling theorem.^23^ The MGS fast version implemented into Focus uses the same calculation technique with fewer ray traces.

Pinnacle^3^ also uses a model based collapsed cone convolution/superposition algorithm to compute dose.^8^ ^,^ ^10^ ^,^ ^12^ Both the Collapsed Cone and Adaptive Convolution models compute TERMA convolved with energy deposition kernels.^21^ For heterogeneous calculations, homogeneous kernels are scaled according to the radiological pathlength using superposition. The Adaptive Convolution algorithm uses the same calculation technique as the Collapsed Cone dose model, but the computation speed is increased by varying the resolution of the calculation grid, based on the gradient of the dose distribution.

### D. Modeling correction

In order to evaluate the accuracy of the treatment planning systems' use of heterogeneity corrections, it is important to equalize any differences in the central axis percent depth dose fit between each model and the actual percent depth dose data. We introduce a modeling correction to account for this. An exact match of data measured for a given beam to the modeled data for that beam is not feasible, therefore this correction will ensure that the beam data is correct for each field without the use of heterogeneity corrections. In addition, the modeling correction will force the beams modeled with CMS to match those modeled with ADAC, before the application of heterogeneity corrections.

Figure 1 illustrates the need for this correction. Percent depth dose a shows a model that could be acceptable at 1.5% greater than the measured percent depth dose for the given field size. Percent depth dose *β* demonstrates a modeled PDD that could be acceptable at 1.5% less than the measured percent depth dose for same field size. While both models (*α* and *β*) are within acceptable limits of the standard, they are significantly different from each other. The modeling correction attempts to eliminate such differences before the application of heterogeneity corrections, thus a balanced comparison can be made between algorithms undiluted by how well the user can force the model to fit specific data.

To generate this correction, the effective depth for each investigated field was determined. With heterogeneity corrections turned off, monitor units calculations were generated on the treatment planning systems for an infinite water phantom with the appropriate field size, effective depth, and energy. These monitor units were compared to our standard (tabled data) calculations for an infinite homogeneous phantom. Differences between the monitor units from the standard and the treatment planning system were calculated to determine the correction to be applied to the monitor units delivered to the anthropomorphic phantom.

### E. Anthropomorphic phantom measurements

Holes were available in the anthropomorphic phantom, which allowed placement of the diode at the isocenter of each of the pelvis, head and neck, lung, and chest wall sites. The monitor units generated from each treatment planning algorithm were delivered. The dose delivered to the diode was compared with the intended 100 cGy.

The setup of the pelvis creates the least challenging geometry. Each beam encounters little heterogeneity, with the isocenter in the center of the phantom. Irradiation of the head and neck presents a slightly more complex situation, with little heterogeneity, but involving missing tissue, reducing the presence of side scattered radiation. The lung section of the phantom introduces a significant amount of heterogeneity. In particular a majority of the LPO beam passes through low density “lung” rather than tissue with almost no buildup. The chest wall exhibits the most complicated geometry. The point of calculation resides at the interface of the lung and the chest wall tissue. In addition, a substantial fraction of the beam is flashed over the surface of the phantom, creating both a lack of side scatter and a significant amount of heterogeneity.

## III. Results

### A. Diode evaluation

Diode response at various depths was determined for 4, 6, 10, and 18 MV. The standard percent depth dose was divided by the average diode reading at each given depth to generate the depth‐dependent output in Table II (a dose of 100 cGy was given to $D_{\max}$).

**Table II:** Depth‐dependent calibration (cGy/nC) for the diode detector.

Depth (cm)	4 MV	6 MV	10 MV	18 MV
$D_{\max}$	0.678	0.669	0.681	0.662
5.0	0.694	0.693	0.693	0.675
10.0	0.702	0.697	0.691	0.678
15.0	0.711	0.705	0.691	0.680
20.0	0.720	0.709	0.690	0.682

### B. Modeling differences

A modeling correction factor, which eliminates the error between the model and the standard set of data, was determined. Table III illustrates an example of the correction factors generated for the pelvis data. Application of the correction factors allows comparison of each algorithm to be independent of modeling errors, which were generally 2% or less.

**Table III:** Modeling correction factors for the pelvis geometry.

Energy	Field	CMS‐FFT	ADAC–adaptive convolution
18 MV	AP	0.991	0.983
18 MV	LT LAT	0.992	0.992
18 MV	LAO	0.984	1.000
10 MV	AP	1.000	0.969
10 MV	LT LAT	1.000	0.965
10 MV	LAO	0.993	1.007

### C. Phantom measurements

The monitor unit settings for irradiation of the anthropomorphic phantom were determined by applying the correction factors mentioned in the previous section (modeling error corrections) to the monitor units generated from the treatment planning algorithm. See Eq. (1),

(1) $${\text{MU}}_{\text{delivered}}={\text{MU}}_{\text{tpa}}\times {\text{Output}}_{\text{depth}}\times {\text{CF}}_{\text{model}}.$$

**Figure 7:**
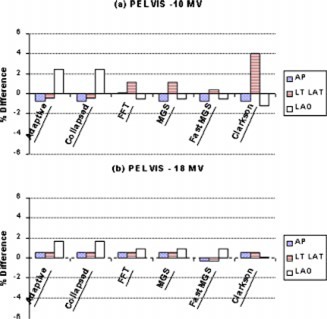
(Color) Comparison (% difference) of the doses delivered to the pelvis phantom with (a) 10 MV and (b) 18 MV.

**Figure 8:**
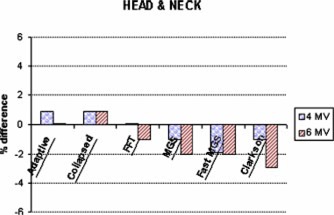
(Color) Comparison (% difference) of the doses delivered to the head and neck phantom.

**Figure 9:**
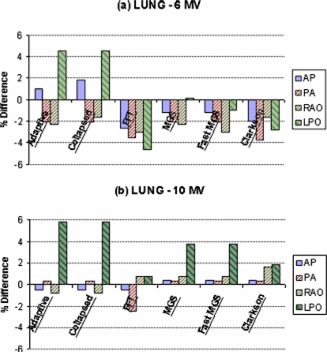
(Color) Comparison (% difference) in dose delivered to the lung phantom with (a) 6 MV and (b) 10 MV.

**Figure 10:**
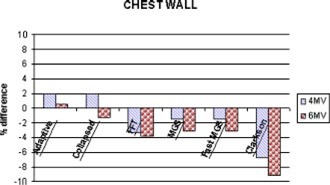
(Color) Comparison (% difference) of the doses delivered to the chest wall phantom.

Figures 7–10 present the percent difference of the calculated dose from each algorithm compared to the dose delivered to each of the four clinical sites. The charts are presented in increasing order of complexity: pelvis, head and neck, lung, and chest wall. A measurement error of ±2% is present, accounting for fluctuations in axial directional response of the silicon diode.^19^

The doses delivered to the pelvis showed little difference between the algorithms' monitor unit calculations [see Figs. 7(a) and 7(b)]. The accuracy of each algorithm applied to the pelvis fell within the acceptable criterion (±4%) outlined by Van Dyk^20^ for anthropomorphic phantoms with heterogeneities. Each algorithm's predicted dose fell within 2% of the measured dose with the exception of the lateral 10 MV using Clarkson (4%). Results from irradiation of the head and neck produced similar results (see Fig. 8). Measured doses fell to within 2% of predicted values with the exception of the Clarkson algorithm for the 6 MV beam. Calculation differences between the algorithms were clinically insignificant.

Analysis of the lung phantom data showed more variation. The data for the AP, PA, and RAO fields showed no clinically significant differences between the calculations of each algorithm [see Figs. 9(a) and 9(b)]. Calculations for each algorithm fell within the 4% criteria for acceptability.^20^ Delivered doses [Figs. 9(a) and 9(b)] for the LPO field ranged from 4.6% low for FFT with 6 MV to 5.8% high for both of ADAC's algorithms with 10 MV.

There is no clinically significant difference between the number of monitor units calculated by the algorithms for the chest wall with the exception of the Clarkson (see Fig. 10). Each algorithm produced results within 4% of the intended dose, however, the doses delivered to the chest wall with the monitor units obtained from the Clarkson calculations were 6.8% too low for 4 MV and 9.1% too low for 6 MV.

Comparison between CMS's superposition and fast superposition showed no statistical differences (p<0.05). Comparisons between ADAC's two algorithms also showed no statistical difference (p<0.05).

## IV. Discussion and Conclusions

Variability for the pelvis geometry and head and neck geometry could be attributed to roundoff and measurement error. Both situations presented little heterogeneity and almost no missing tissue.

Variations in the calculations for the lung fields showed little differences between the algorithms for the AP, PA, and the RAO fields. Calculations for these beams encountered a small percentage of low density lung, but the calculation point has sufficient tissue buildup to provide electronic equilibrium. All calculations for these beams fell within the ±4% criteria of acceptability. In contrast, the considerable amount of low density lung in the LPO field reduced the accuracy of the calculations. Given the lack of buildup to provide electronic equilibrium, the accuracy of the calculations for the LPO beam fell within reasonable limits. The variability between 6 and 10 MV results for this beam can be attributed to the considerable dose gradient associated with the measurements and calculations being performed in the buildup region. Typically beam models are forced into agreement at depths beyond $D_{\max}$ (depth of maximum dose) with known error accepted at depth up to $D_{\max}$. These known tolerances contribute to the greater errors associated with this beam.

The Clarkson calculations for the chest wall illustrated the algorithm's inability to accurately handle situations with a significant portion of missing tissue. The calculations neglected the missing scattered radiation and hence did not predict enough monitor units.

The accuracy of the six algorithms' ability to predict monitor units for a variety of clinical situations were acceptable with the exception of using Clarkson for the chest wall. Certain algorithms were slightly better under certain conditions, but no algorithm clearly stood out for all situations. ADAC's convolution algorithms and CMS's FFT, Superposition and Fast Superposition algorithms produce clinically acceptable monitor unit calculations. The Clarkson calculated monitor units are reliable for simple situations, but discretion should be used when using monitor unit calculations for a complex geometry.
